# Cross-disciplinary communication between oral and gut microbiota in head and neck cancer

**DOI:** 10.3389/fonc.2026.1740060

**Published:** 2026-02-04

**Authors:** Xinhua Lin, Hanbin Qin, Zhonglu Liu, Xin Zhao, Xuexia Liu, Hua Zhang

**Affiliations:** 1 School of Clinical Medicine, Shandong Second Medical University, Weifang, China; 2 Department of Otorhinolaryngology, Head and Neck Surgery, Yantai Yuhuangding Hospital, Qingdao University, Yantai, China; 3 Shandong Provincial Clinical Research Center for Otorhinolaryngologic Diseases, Yantai, China; 4 Shandong Provincial Key Laboratory of Neuroimmune Interaction and Regulation, Yantai, China; 5 Shandong Engineering Research Center for Precision Diagnosis and Treatment of Airway Diseases, Yantai, China; 6 Yantai Key Laboratory of Otorhinolaryngologic Diseases, Yantai, China; 7 Yantai Clinical Research Center for Otorhinolaryngologic Diseases, Yantai, China; 8 Shandong Stem Cell Engineering Technology Research Center, Central Laboratory, Affiliated Yantai Yuhuangding Hospital of Qingdao University, Yantai, China

**Keywords:** gut microbiota, HNC, oral microbiota, oral-gut axis, tumor

## Abstract

Head and neck cancer (HNC) is a prevalent malignancy with a rising global incidence. While traditional risk factors such as tobacco use and viral infections are well-established, the dysbiosis of oral and gut microbiota has recently emerged as a pivotal contributor to HNC pathogenesis. The oral-gut axis serves as a critical conduit for bidirectional microbial crosstalk, facilitated by bacterial translocation, metabolic exchange, and immune modulation, collectively fostering a pro-tumorigenic microenvironment. Key oral pathogens, including *Fusobacterium nucleatum* and *Porphyromonas gingivalis*, exacerbate chronic inflammation, promote immune evasion, and activate oncogenic signaling pathways such as Wnt/β-catenin, MAPK/ERK, and PD-1/PD-L1. In parallel, gut dysbiosis influences HNC progression by altering the production of microbial metabolites, including short-chain fatty acids, bile acids, and tryptophan derivatives, which systemically regulate inflammation and anti-tumor immunity. Growing evidence also implicates the microbiota in modulating responses to radiotherapy, chemotherapy, and immunotherapy. Therapeutic strategies targeting the oral-gut axis, including probiotics and antimicrobial peptides, hold promise for alleviating treatment-induced mucosal injury and improving therapeutic outcomes. Nonetheless, significant challenges persist, including elucidating network-level microbial interactions, validating robust biomarkers, and advancing these findings into clinical practice. Future multidisciplinary collaborations are essential to fully leverage the oral-gut microbiota axis for precision oncology in HNC.

## 1 Introduction

HNC encompasses a group of malignancies arising from the mucosal epithelium of the upper aerodigestive tract, ranking as the seventh most common cancer worldwide with an annual incidence exceeding 660,000 cases (1). While traditional risk factors like tobacco, alcohol, and viral infections (e.g., HPV, EBV) are well-recognized, a growing body of evidence highlights the microbiome as a key player in HNC pathogenesis.

The human microbiome, particularly the communities residing in the oral cavity and gut, constitutes a complex ecosystem that exerts systemic influence on host physiology and disease. The oral cavity, hosting the body’s second most diverse microbial population, is crucial for maintaining local and systemic homeostasis. Dysbiosis in this community is implicated in conditions ranging from dental caries to systemic diseases, including cancer. Similarly, the gut microbiota, the largest assemblage of microorganisms in the body, is integral to metabolism, immunity, and barrier function. Its disruption is associated with an elevated risk of various malignancies, with epidemiological studies attributing up to 20% of cancers to microbial factors that promote genomic instability and aberrant cell signaling (2).

**Figure 1:**
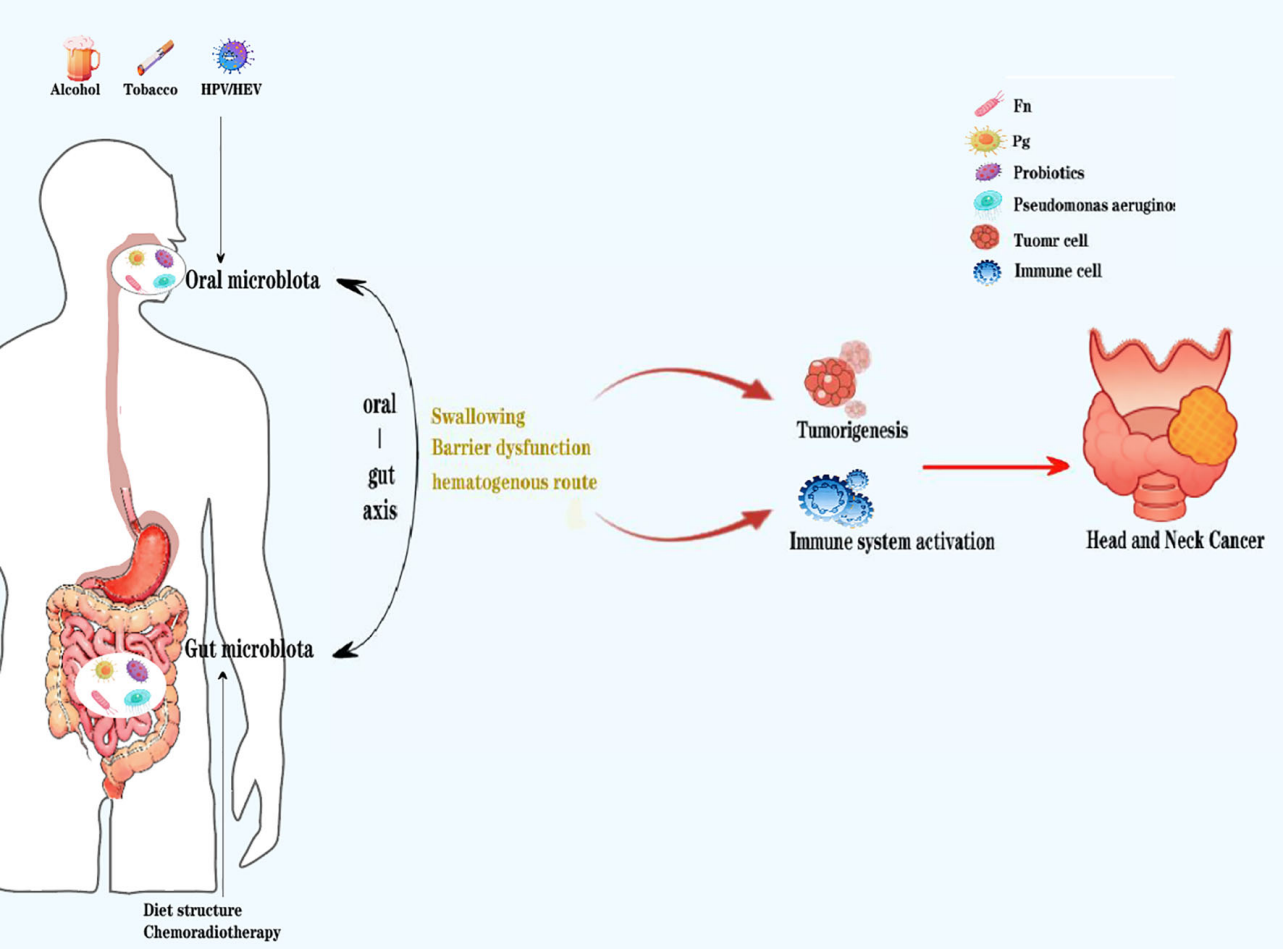
When the oral and gut microenvironment changes due to external factors (such as smoking, alcohol consumption, HPV/HIV virus, dietary structure, chemotherapy, etc.), the microbial community structure is prone to imbalance, mainly manifested as an increase in pathogenic bacteria such as Pg and Fn, and a decrease in beneficial bacteria such as probiotics. Due to damage to the oral and intestinal mucosal barrier, the microbiota undergoes significant enhancement of local oral and gut microbiota migration through swallowing and blood exchange, thereby disrupting the homeostasis of the systemic immune microenvironment, promoting tumor cell growth, and ultimately creating conditions for the occurrence and development of head and neck tumors.

This has led to the conceptualization of the “oral-gut axis,” a bidirectional communication network facilitated by anatomical continuity. Under physiological conditions, robust barrier functions maintain distinct microbial niches. However, upon barrier dysfunction, microbial translocation, metabolic exchange, and immune crosstalk create a pro-carcinogenesis feedback loop (3). For example, swallowing in HNC patients can disseminate oral pathogens to the gut, altering its ecology, while gut-derived metabolites can systemically influence the oral cancer microenvironment. Thus, elucidating the mechanisms of the oral-gut axis in HNC offers a transformative, microecology-based perspective for developing novel diagnostic, preventive, and therapeutic strategies (**Table 1**; **Figure 1**).

**Table 1:** The role of oral gut microbiota in the occurrence of HNC.

Microbial community name	Classification of phyla	Beneficial/Harmful	Sampling method	Mechanism of action	Associated cancer types	References
*Prevotella*	Bacteroidetes	harmful	saliva	Generate virulence factors (lipoteichoic acid, peptidoglycan, adhesin pili, and LPS)	oral squamous cell carcinoma	(8)
*Pseudomonas aeruginosa*	Proteobacteria	harmful	Oral tissue	Causing DNA breakage in epithelial cells, promoting inflammation through their flagella, lipopolysaccharides, and ExoU cell toxins, the latter of which can activate the nuclear factor kB pathway and lead to IL-8 secretion;	oral squamous cell carcinoma	(4)
*Fusobacterium nucleatum*	Fusobacteria	harmful	Oral tissue, saliva	Inducing the expression of B defense factor 2 in oral epithelial cells, leading to the release of pro-inflammatory cytokines such as IL-6 and IL-8. Through B defense factor 2, degranulation of mast cells also occurs, leading to histamine release and further stimulating the production of B defense factor	oral squamous cell carcinoma, HNC	(4, 7)
*Porphyromonas gingivalis*	Bacteroidetes	harmful	Oral tissue'saliva	Inhibition of cytochrome C, downregulation of caspase 3 activity, secretion of anti-apoptotic enzyme nucleoside diphosphate kinase that leads to inhibition of P2X receptors on epithelial cells, and upregulation of microRNA203 to prevent cell apoptosis, while upregulation of anti-apoptotic genes Bcl-2 and Survivors	oral cancer, HNC	(4, 7, 8)
*Actinobacterium*	Actinobacteria	harmful	feces	Upregulation of CCL20 production in oral cancer cell line	oral cancer	(3, 4)
*Rod-shaped bacteria*	Actinobacteria	harmful	saliva	Effectively metabolize ethanol into acetaldehyde, disrupt DNA repair, and increase cancer risk	HNC	(3)
*Streptococcus mutans*	Firmicutes	harmful	saliva	Effectively metabolize ethanol into acetaldehyde, disrupt DNA repair, and increase cancer risk	HNC	(3)
*Bifidobacterium*	Actinobacteria	harmful	saliva, feces	Regulating gene transcription related to inflammation and cell apoptosis, and inhibiting adverse events; Increase the number and activity of immune cells, and enhance the efficacy of immune checkpoint inhibitors targeting CTLA-4 and PD-L1	HNC	(3, 9, 10)
*Pseudomonas genus*	Clostridium difficile phylum	harmful	saliva'Oral tissue	Enrichment under chronic stress conditions promotes dysfunction of the oral and intestinal barrier, induces changes in the metabolome (such as elevated levels of plasma kynurenine), promotes CD8+T cell depletion by activating aromatic hydrocarbon receptors (AhR), and drives tumorigenesis; Its flagella, lipopolysaccharides, and ExoU cell toxins can induce inflammation. ExoU can also activate the nuclear factor kappa B pathway and induce IL-8 secretion. Its epithelial cell DNA breaks, enhances invasion and metastasis ability	oral cancer, HNC	(11)
*Veillonella*	Firmicutes	harmful	saliva, feces	Accumulation under chronic stress, involvement in oral microbiota dysbiosis, disruption of host barrier function, and synergistic promotion of inflammatory state in the tumor microenvironment	HNC	(11)
*Lactobacillus*	Firmicutes	beneficial	saliva, feces	Generate SCFA and other valuable metabolites, alter intestinal pH, produce antibacterial components, and regulate the brain skin gut axis. Treat periodontitis and improve mild sadness symptoms in adults. Reduce the side effects of cancer treatment (such as diarrhea and OM)	HNC	(8, 10)
*Oral Streptococcus pneumoniae*	Firmicutes	harmful	saliva	Generate different acids, resulting in low pH, which helps with proliferation and transfer processes	oral cancer	(8)
*Candida albicans*	Ascomycota	harmful	mouthwash, saliva and oral swabs	Generate nitrosamines, carcinogenic substances	oral cancer	(8)
*Streptococcus gordonii, Streptococcus parahaemolyticus, and Streptococcus salivarius*	Streptococcus genus	harmful	saliva	Generate acetaldehyde and display alcohol dehydrogenase activity	oral cancer	(4)
*Trichoderma*	Ascomycota	harmful	feces	Participate in the imbalance of gut microbiota structure, which may promote tumor progression by affecting host metabolism or immune regulation	nasopharyngeal carcinoma	(12–14)
*Klebsiella*	Proteobacteria	harmful	Feces, saliva	Participate in the imbalance of gut microbiota structure, which may promote tumor progression by affecting host metabolism or immune regulation	nasopharyngeal carcinoma, oral cancer	(12–14)
*Clostridium ramosum*	Firmicutes	harmful	feces	Metabolites stimulate cells to secrete 5-hydroxytryptophan, leading to an increase in plasma 5-hydroxytryptophan levels and promoting the development of nasopharyngeal carcinoma	nasopharyngeal carcinoma	(12–14)

## 2 Characteristics and interactions of oral and gut microbiota

### 2.1 Oral microbiota

The oral cavity is a primary site for microbial colonization, hosting a complex and diverse microecological environment. Distinct niches, such as the tooth surface, gingival sulcus, saliva, tongue dorsum, and oral mucosa, provide unique habitats for a wide array of microorganisms. Metagenomic studies have revealed that the microbial communities in healthy individuals are predominantly composed of genera such as *Streptococcus*, *Diplococcus*, *Veillonella*, *Haemophilus*, *Neisseria*, *Porphyromonas*, *Fusobacterium*, *Actinomyces*, and *Prevotella* (3). These microorganisms maintain a dynamic equilibrium through nutritional competition and metabolic cooperation, thereby preserving oral microecological stability under physiological conditions.

However, external factors—including poor oral hygiene, diminished host immunity, and radiotherapy or chemotherapy—can disrupt the oral microenvironment, leading to microbial dysbiosis. This imbalance is characterized by the overgrowth of pathogenic bacteria and a decline in beneficial species, increasing the risk of oral diseases. Furthermore, host-specific factors such as age, behavioral habits, salivary composition, and immune status continuously reshape the oral microbiome over time. For instance, the abundance of *Bacteroidetes* (mainly *Prevotella*) and *Veillonella* tends to increase with age, whereas bacteria such as Granulicatella show a decreasing trend (4).

In common oral diseases like periodontitis and dental caries, shifts in microbial composition are central to pathogenesis. Overall dysbiosis, coupled with the overgrowth of pathogens such as *Prevotella*, *Fusobacterium nucleatum* (Fn), *Porphyromonas gingivalis* (Pg), *Tannerella forsythia*, and *Treponema denticola*, can trigger host immune responses, leading to gingival inflammation and disease progression. Notably, certain periodontal pathogens, including Pg, have been associated with oral precancerous lesions and oral squamous cell carcinoma (OSCC), potentially through mechanisms involving chronic endogenous infection and modulation of host inflammatory responses (5).

Beyond local oral diseases, oral microecological imbalance is also closely linked to a range of systemic conditions, including Alzheimer’s disease, diabetes, adverse pregnancy outcomes, and multiple malignancies such as oral, gastrointestinal, lung, breast, prostate, and uterine cancers (6). These associations underscore the profound impact of oral microbiota on overall health.

### 2.2 Gut microbiota

The gut microbiota is a complex microbial community predominantly composed of anaerobic bacteria, with major phyla including Bacteroidetes, Firmicutes, Actinobacteria, and Proteobacteria. Its composition, structure, and functional diversity are shaped by host genetics, dietary patterns, lifestyle, and medication use. As the largest microecosystem in the human body, the gut microbiota profoundly influences intestinal physiology and pathology through mechanisms such as metabolite synthesis and maintenance of mucosal homeostasis (5).

Anatomically, the intestinal barrier—comprising the mucus layer, epithelial cells, and tight junctions—serves as a “gatekeeper,” maintaining internal stability by selectively permitting nutrient absorption while restricting the passage of pathogens and harmful metabolites. Impairment of this barrier, leading to increased permeability, can trigger chronic inflammation, allergic responses, and autoimmune disorders.

Gut dysbiosis has been implicated in numerous diseases, including inflammatory bowel disease, type 2 diabetes, atherosclerosis, obesity, and neuropsychiatric conditions. Recent studies have identified characteristic gut microbial profiles in patients with various cancers, particularly those of the digestive tract, suggesting a potential role for gut microecological imbalance in tumorigenesis (6). Beneficial genera such as *Lactobacillus* and *Bifidobacterium* are generally associated with anti-inflammatory effects, whereas opportunistic pathogens like *Escherichia coli* and *Shigella* are linked to pro-inflammatory states.

Notably, the gut microbiota exhibits high sensitivity to dietary and environmental influences. Through the fermentation of dietary components, gut microbes produce essential metabolites—including short-chain fatty acids (SCFAs), tryptophan derivatives, and bile acids—that regulate gene expression, immune cell function, and systemic immune balance. For instance, SCFAs derived from dietary fiber not only lower the luminal pH but also promote polyamine synthesis, thereby reinforcing intestinal homeostasis. Probiotics, including bifidobacteria and lactobacilli, contribute to host health by aiding nutrient digestion, inhibiting pathogen colonization, and degrading toxins. Additionally, gut microbes participate in the synthesis of vitamins such as vitamin K2 and B vitamins, with recent evidence suggesting that some strains may even produce vitamin C (6).

Conversely, poor dietary habits—such as high intake of sugar, fat, and protein—may promote the expansion of bile-tolerant microorganisms. These bacteria can produce genotoxic secondary bile acids and pro-inflammatory metabolites (e.g., hydrogen sulfide), creating a favorable microenvironment for colorectal carcinogenesis. Proposed mechanisms include direct DNA damage, activation of pro-tumor signaling pathways, production of oncogenic metabolites, and suppression of anti-tumor immunity. A 2021 study by Deng et al. further revealed an increased abundance of potential pathobionts (e.g., *Prevotella* and *Actinomyces*) and a reduction in SCFA-producing beneficial bacteria (e.g., *Bacteroides*) in cancer patients (7). Such dysbiosis is often accompanied by elevated endotoxin production and impaired barrier function, potentially leading to systemic inflammation that supports tumor development. Importantly, SCFAs like butyrate not only exert anti-inflammatory effects but also enhance intestinal barrier integrity, highlighting the therapeutic potential of modulating gut microbiota in cancer prevention and treatment. 

### 2.3 Pathways of microbial crosstalk

Anatomically connected via the gastrointestinal tract, the oral and gut compartments form a continuous system that facilitates microbial transit. Beyond physical continuity, they interact through saliva and digested food materials. The gastrointestinal barrier exhibits selective permeability, ensuring efficient nutrient absorption while defending against pathogenic invasion (15).

Studies indicate that oral-to-gut microbial translocation occurs primarily through two pathways: the “enteric route” (16, 17), in which oral bacteria traverse the esophagus and stomach via swallowing—with species such as Fn demonstrating gastric acid resistance—and the “hematogenous route” (18), wherein bacteria enter the bloodstream through compromised oral mucosal barriers and disseminate to distant sites including the intestine.

Under physiological conditions, defense mechanisms such as gastric acid, intestinal peristalsis, and digestive enzymes collectively form an efficient “microbial clearance system” that eliminates most orally ingested microbes, maintaining gut microbial homeostasis. However, oral dysbiosis or gastrointestinal barrier impairment can disrupt this fine-tuned regulation, leading to abnormal colonization of oral-derived microbes in the gut. This translocation has been linked to chronic inflammatory conditions such as ileitis, colitis, and colorectal cancer (19). For example, the oral pathobiont Fn can proliferate significantly under poor oral hygiene. Oral administration of Fn in a mouse model of experimental colitis was shown to induce inflammatory responses and tumor formation in the small intestine and colon (20). This microbial crosstalk is bidirectional. Li et al. demonstrated through *in vitro* studies that gut microbes can translocate retrograde to the oral cavity under conditions of gastrointestinal dysfunction. Clinical observations have identified abnormal colonization of typical oral commensals such as *Haemophilus* and *Veillonella* in the intestinal mucosa of patients with inflammatory bowel disease. Notably, this cross-site colonization begins early in life: *Bifidobacterium*, a dominant gut genus, has been detected in the oral fluid of newborns. In contrast, elderly individuals exhibit a higher prevalence of oral-characteristic bacteria (e.g., *Porphyromonas*, *Fusobacterium*, and *Pseudobacterium*) in the gut compared to healthy adults. Thus, the oral–gut axis represents a critical dimension of human health and is increasingly recognized as a key pathway in disease pathogenesis (3).

## 3 Dysbiosis characteristics in HNC

### 3.1 Oral microbiota in HNC

The global incidence of HNC continues to rise, posing a significant public health challenge. Beyond established risk factors such as tobacco use, alcohol consumption, and viral infections, growing evidence indicates that specific oral microbial communities play a critical role in HNC pathogenesis (21). Although the overall composition of the oral microbiota in HNC patients shares similarities with that of healthy individuals, significant differences emerge at finer taxonomic resolutions. At the phylum level, Firmicutes often dominate. At the genus level, notable decreases are observed in the abundance of *Pseudomonas*, *Acinetobacter*, and *Shigella*. Conversely, at the species level, Fn and Pg are consistently enriched and have become a major research focus due to their potential involvement in HNC development through diverse mechanisms.

**Figure 2:**
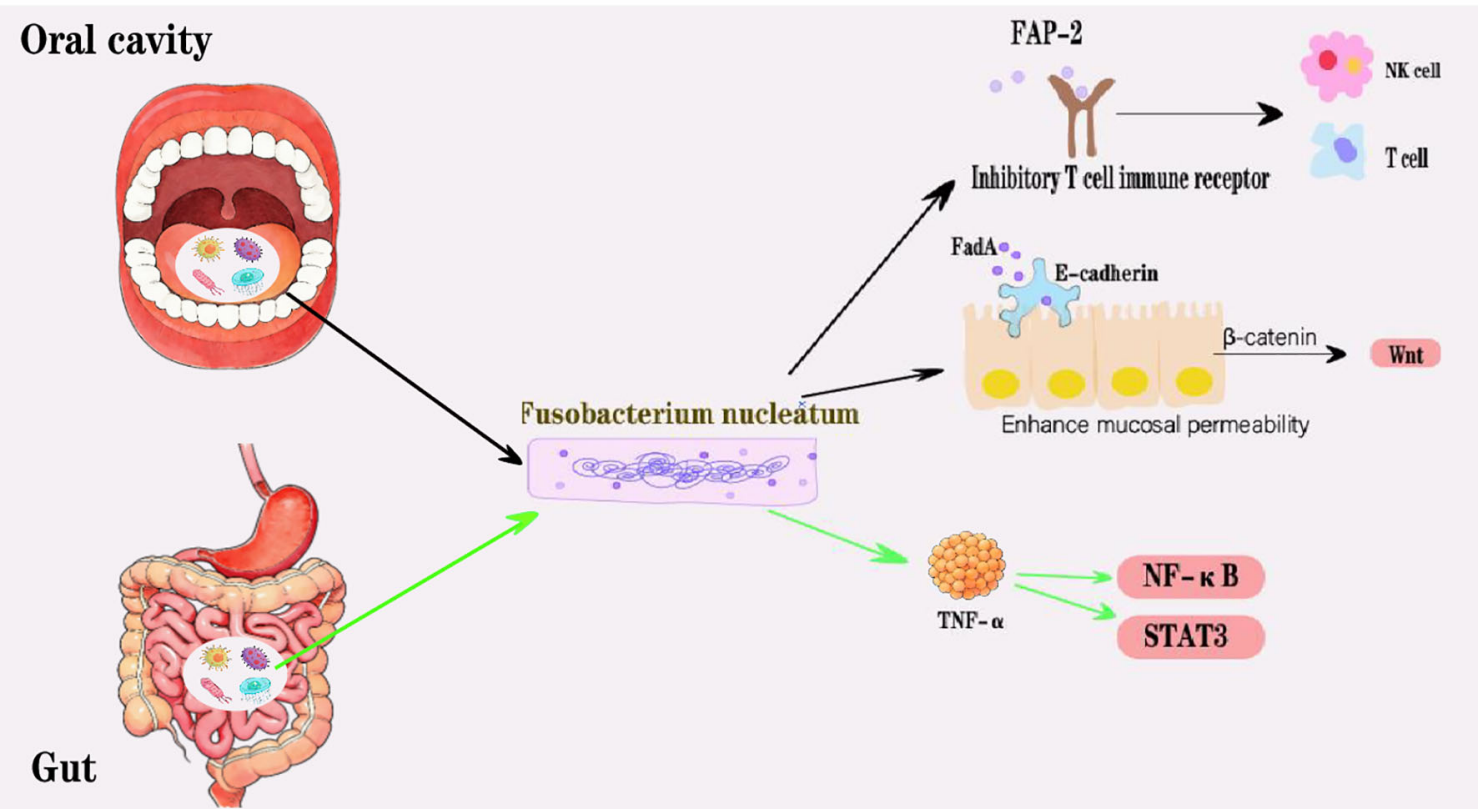
In the oral cavity, Fn’s adhesion protein FadA can bind to E-cadherin on the surface of epithelial cells and inactivate it, thereby enhancing mucosal permeability. Simultaneously, the intracellular free breeacell will also increase, activating the Wnt signaling pathway. Furthermore, Fn proteins (such as FAP-2) can block and reduce the activity of NK cells and T cells by binding to inhibitory T cell immune receptors. In intestinal tissues, Fn can promote the production of TNF-uc activate the NF-iv signaling and STAT3 signaling pathways. Fn may achieve the invasion and metastasis of HNC through the above mechanism.

Fn is not only associated with oral conditions like chronic periodontitis but also contributes to HNC progression. Salem et al. proposed that Fn can induce the expression of β-defensin 2 in oral epithelial cells—a molecule implicated in the pathogenesis of oral lichen planus and tongue cancer—thereby stimulating the production of potent pro-inflammatory cytokines such as interleukin 6 (IL-6) and interleukin 8 (IL-8). This process can trigger mast cell degranulation and histamine release, establishing a self-perpetuating cycle of chronic inflammation. This inflammatory microenvironment is characterized by the sustained release of pro-inflammatory cytokines, immune cell recruitment, and progressive angiogenesis (4). The Fn adhesin FadA binds to E-cadherin on epithelial cells, inactivating it and enhancing mucosal permeability. This interaction also leads to the accumulation of free β-catenin, activating the Wnt signaling pathway and driving cellular proliferation. Furthermore, Fn proteins such as Fap-2 can bind to inhibitory immune receptors on T cells and natural killer (NK) cells, blunting their anti-tumor activity (22). Fn infection has also been linked to increased matrix metalloproteinase 9 and matrix metalloproteinase 13 activity, suggesting a potential role in basement membrane degradation and tumor invasion (23). Collectively, Fn infection is associated with high-grade dysplasia, lymph node metastasis, and poor prognosis in HNC (**Figure 2**).

Pg has been identified as an independent risk factor for oral cancer-related mortality (24).This pathogen activates Toll-like receptor 2 (TLR2) and Toll-like receptor 4 (TLR4), inducing the release of IL-8 and other pro-inflammatory mediators. Intracellularly, Pg suppresses apoptosis through multiple mechanisms, including inhibition of cytochrome c, downregulation of caspase-3 activity, secretion of anti-apoptotic nucleoside diphosphate kinase, and upregulation of specific microRNAs (25).It also upregulates the expression of anti-apoptotic genes such as Bcl-2 and Survivin (26) (308–96). The gingipains produced by Pg activate and mature matrix metalloproteinase 9, facilitating basement membrane degradation and OSCC metastasis (308–98). Moreover, Pg can mediate T cell inhibition by upregulating programmed death-ligand 1 (PD-L1), enabling OSCC cells to evade immune surveillance, and can induce the expression of B7-H1 and B7-dendritic cell receptors, leading to apoptosis of activated T cells (27). Pg has also been shown to induce epithelial-mesenchymal transition primarily through its FimA adhesin, affecting p53, phosphatidylinositol 3-kinase, and cyclin pathways to accelerate cell cycle progression (28). Additionally, Pg reduces the activity of plakophilin, a key protein for epithelial integrity, thereby promoting metastasis (29). As a major producer of the carcinogen acetaldehyde, Pg also contributes to cancer cell autophagy and chemoresistance (308–103). Intriguingly, Pg may induce HIV reactivation through butyrate production and facilitate viral entry into epithelial cells (30, 31), although potential synergistic roles in HNC pathogenesis in HIV-positive patients require further investigation.

Although less frequently detected in HNC, *Pseudomonas aeruginosa* has attracted attention due to its potential carcinogenicity. This bacterium can induce DNA breaks in epithelial cells (32) and promote tumor cell invasion and metastasis. Its pathogenicity is linked to virulence factors such as flagella and lipopolysaccharides, which directly provoke inflammation, and the ExoU cytotoxin, which activates the NF-κB signaling pathway and stimulates IL-8 secretion, fostering a pro-tumor microenvironment (33). The role of fungi in OSCC progression is also increasingly recognized. Elevated abundance of oral *Candida* is positively correlated with the severity of oral epithelial dysplasia (34). *Candida albicans*, in particular, can provide colonization support for pathogenic bacteria like Pg (35), damage keratinocytes by secreting aspartic proteases and inducing endocytosis, and degrade the basement membrane protein laminin in an acidic pH environment. This suggests that the acidification of the OSCC microenvironment may synergize with C. albicans to enhance tissue damage (36).

The dynamic composition of the oral microbiota is also closely linked to HNC treatment responses and postoperative recovery. Chemoradiotherapy often increases the abundance of oral opportunistic pathogens, such as *Prevotella* and *Fusobacterium*, elevating the risk of early-onset radiation-induced oral mucositis (RIOM). A lower abundance of Streptococcus is associated with a shorter time to the onset of severe oral mucositis (OM) (37). In a cohort study of patients undergoing head and neck free flap reconstruction, surgical site infection (SSI) within 30 days post-surgery occurred in 67 patients (13.3%). Pathogens derived from the oral flora were identified in 75% of these cases, including *Gram-negative bacilli* (44%), methicillin-resistant *Staphylococcus aureus* (MRSA, 20%), and methicillin-sensitive *Staphylococcus aureus* (MSSA, 16%). From a diagnostic perspective, pioneering work by Mager et al. has advanced the non-invasive detection of HNC. Their saliva-based studies identified characteristic expression patterns of *Prevotella* melaninogenica and *Streptococcus* mitis in OSCC patients. A diagnostic model based on these bacterial biomarkers achieved a predictive accuracy of up to 80% (38), providing a crucial foundation for novel, non-invasive diagnostic strategies.

In summary, the oral microbiome plays a significant role in the development, treatment, and diagnosis of HNC. Future research focusing on microbiome-based interventions may open new avenues for precision medicine in HNC.

### 3.2 Gut microbiota in HNC

As the largest and most complex microbial ecosystem in the human body, the gut microbiota plays a crucial role in HNC pathogenesis and progression. Clinical interventions, including the side effects of chemoradiotherapy, dietary changes, and antibiotic use, can significantly alter its composition and function (4). The gut microbiota influences host physiology by participating in dietary metabolism, synthesizing essential nutrients like vitamins, and generating key bioactive metabolites such as SCFAs. SCFAs exert anti-inflammatory effects by inducing interleukin 10 (IL-10) release and suppressing pro-inflammatory cytokines like IL-6 and tumor necrosis factor-s (TNF-r-. They also activate G-protein-coupled receptors (GPCRs) on epithelial and immune cells, modulating broad immune-inflammatory responses and even contributing to the inactivation of head and neck squamous cell carcinoma (HNSCC) cells (39). The gut microbiota may influence HNC through multiple pathways [67: some bacteria can directly cause DNA damage or activate pro-tumor signaling pathways, while others produce cancer-promoting metabolites or suppress anti-tumor immunity. Diet, a major modulator of the gut microbiota, is also linked to HNC risk. SCFAs (e.g., butyrate, acetate, propionate), derived from microbial fermentation of dietary fiber, exhibit well-documented anti-inflammatory and anti-cancer properties. Gut dysbiosis can reduce SCFA production, weakening this protective effect and exacerbating systemic inflammation, which may contribute to OSCC development. Dysbiosis also promotes the production of secondary bile acids, which possess pro-inflammatory and cytotoxic properties. These compounds can enter systemic circulation and potentially affect the oral microenvironment, favoring OSCC initiation (40). Furthermore, gut microbes metabolize tryptophan into indole derivatives that regulate immune responses. Dysbiosis can disrupt this process, leading to enhanced inflammation, immune dysregulation, and promotion of OSCC. Therefore, supplementing beneficial microbial metabolites such as SCFAs—either directly or via interventions that stimulate their endogenous production—represents a promising adjuvant strategy to mitigate inflammation, enhance cytoprotection, and potentially reduce OSCC risk.

HNC patients frequently develop OM and xerostomia following radiotherapy, conditions linked to oral and gut dysbiosis. This dysbiosis is characterized by the overgrowth of cariogenic bacteria (e.g., *Streptococcus mutans*, *Lactobacillus*) and opportunistic pathogens (e.g., *Staphylococcus*, *Enterococcus*, *Candida albicans*), alongside a decline in commensals like *Neisseria (*20, 41). *Enterococcus* can enhance microbial proteolytic activity against fibronectin, aggravating radiation-induced mucosal damage and inflammation (4). Radiotherapy can also indirectly compromise the intestinal barrier and induce inflammation. For instance, tongue irradiation in mouse models triggers mitochondrial oxidative stress and activates the NLRP3 inflammasome in the small intestine (39). A study of 47 radiotherapy-treated HNC patients found that most developed grade 2–3 OM, with pre-radiotherapy nutritional support preventing grade 4 OM. Microbiota analysis revealed that a higher OM grade was associated with lower abundance of Proteobacteria. As mucositis worsened, the abundance of most differentially abundant bacteria decreased, suggesting that radiotherapy may influence mucositis development by altering the growth patterns of Proteobacteria. Studies in nasopharyngeal carcinoma have reported a higher abundance of *Clostridium* in patients with mild mucositis and of *Actinomyces* in those with severe mucositis. Similar microbial signatures have been correlated with quality of life in HNC patients, indicating their potential utility as biomarkers for predicting patient outcomes following radiotherapy (9). Beyond radiotherapy, the gut microbiota also modulates chemotherapy efficacy and toxicity. It can enhance or diminish drug efficacy; for example, it can upregulate pro-apoptotic pathways to improve cisplatin response. In the case of gemcitabine, a common treatment for advanced OSCC, γ-Proteobacteria can confer resistance, while co-administration of ciprofloxacin can reverse this resistance and restore drug efficacy. The gut microbiota also influences immunotherapy outcomes. Beneficial taxa such as *Bacteroides* and *Bifidobacterium* can boost immune cell numbers and activity, enhancing the efficacy of immune checkpoint inhibitors targeting CTLA-4 and PD-L1.

In conclusion, the gut microbiota plays a multifaceted role in HNC, profoundly influencing tumor initiation, progression, and treatment response through metabolite production, systemic inflammation, and immune modulation. However, key questions regarding how to precisely modulate the gut microbiota for HNC intervention remain to be answered.

## 4 Oral-gut microbiota axis jointly regulates the occurrence and development of head and neck cancer

As two core microbial ecosystems, the human oral cavity and gut engage in complex bidirectional communication via the “oral-gut microbiota axis” (42). It is estimated that healthy individuals swallow approximately 1.5 liters of saliva daily, thereby transporting millions of oral microbes into the gastrointestinal tract. Approximately 1% of these microbes survive the gastric acid barrier to colonize the gut, contributing significantly to the gut microbiota equilibrium; indeed, about one-third of the gut microbiota in healthy individuals is thought to originate from the oral cavity. However, when the integrity of the oral or gut mucosal barrier is compromised, this finely regulated microbial exchange is disrupted. Enhanced microbial translocation between the two sites can perturb systemic immune homeostasis, thereby creating conditions conducive to the development of oral, gut, and systemic diseases, including head and neck tumors and metabolic syndrome.

**Figure 3:**
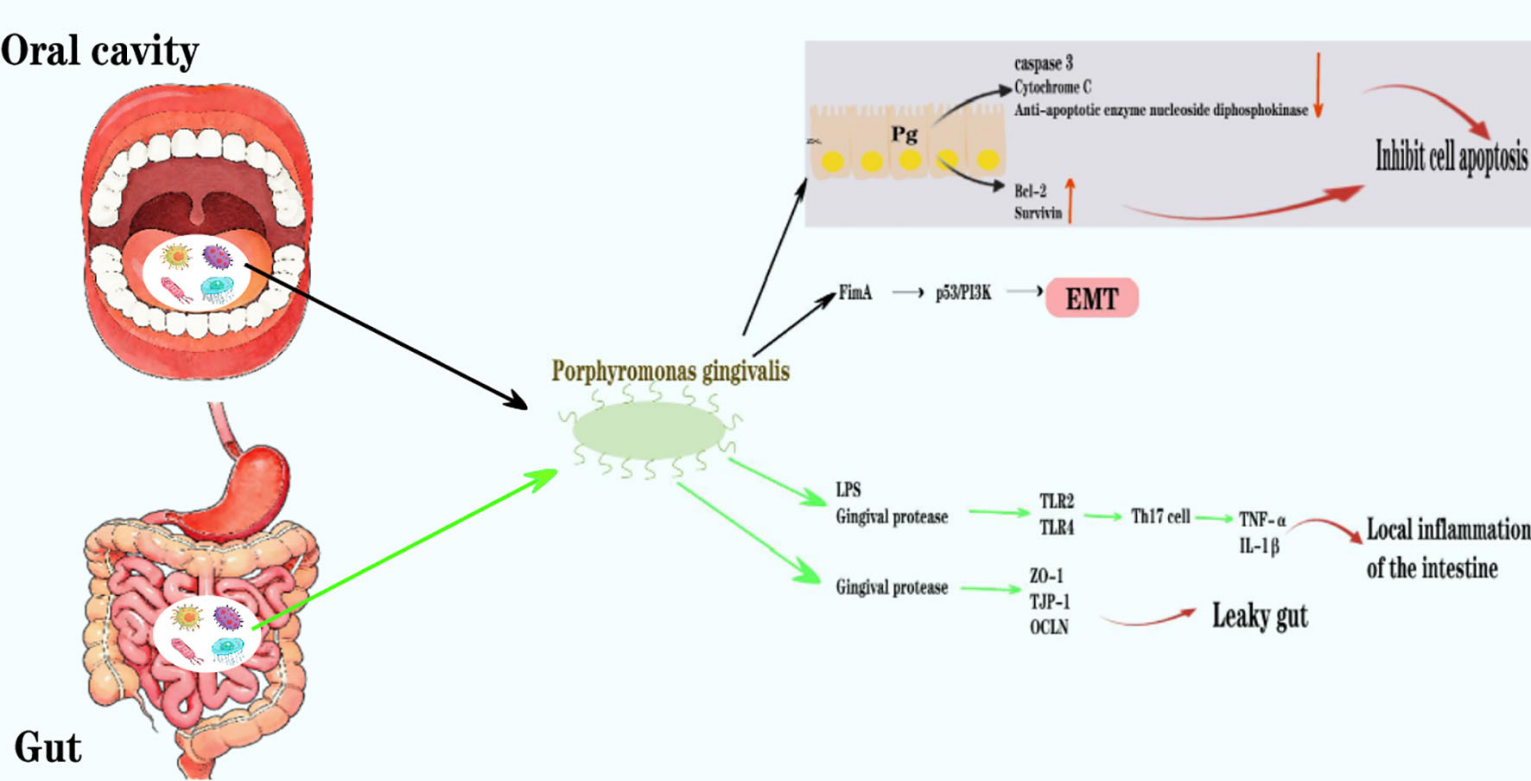
In the oral cavity, Pg inhibits apoptosis through multiple mechanisms, including inhibiting P cell pigment C, downregulating caspase 3 activity, secreting anti-apoptotic enzyme nucleoside diphosphokinase, and upregulating microRNA; Pg can induce epithelial-mesenchymal transition and primarily affects p53, PI3K, and cell cycle protein pathways through its FimA adhesion molecule, thereby accelerating the cell cycle progression. In the intestine, components of Pg such as LPS, gingipain, and extracellular vesicles induce the differentiation of helper T cell 17 (Th17 cells) by stimulating TLR2 and TLR4, thereby promoting the expression of pro-inflammatory factors such as tumor necrosis factor (TNF) and interleukin 1n. This leads to local inflammation in the intestine; the gingipain it produces can directly degrade components of the intestinal mucus layer, disrupting the integrity of the mucus barrier, and simultaneously downregulating the gene expression of tight junction-related proteins (such as occludin 1, tight junction protein 1/TJP-1, and occludin/OCLN) through cleavage, resulting in impaired intestinal epithelial barrier function and increased risk of “intestinal leakage”.

In patients with HNC, the dysbiosis of the oral-gut axis is characterized by distinct microbial shifts: the oral cavity exhibits significant enrichment of pro-inflammatory bacteria such as Fn and Pg, while the gut shows a reduction in beneficial bacteria like *Bifidobacterium*. Oral microbes and their metabolites (e.g., lipopolysaccharide (LPS) gingipains) can disseminate systemically via hematogenous or enteric routes, activating a systemic inflammatory response. Compromised oral mucosal permeability, often resulting from periodontitis, provides a critical gateway for this microbial translocation. Bacteria from periodontal plaques can directly invade periodontal blood vessels or reside within dendritic cells and macrophages, hitchhiking with these immune cells to extra-oral tissues (43). The gut serves as a primary target for colonization. For instance, the abundance of Fn in gut tissue biopsies is twice that found in fecal samples, suggesting a particular affinity for mucosal adhesion. Gut-colonized Fn can promote the production of TNF-α, activate NF-κB and STAT3 signaling pathways, and enhance tumor cell migration and invasion (44). Similarly, Pg can translocate to the gut via swallowing or blood, inducing gut dysbiosis and barrier damage through multiple mechanisms. First, Pg components such as LPS, gingipains, and extracellular vesicles can stimulate Toll-like receptors (TLR2, TLR4), promote the differentiation of Th17 cells, and trigger the expression of pro-inflammatory factors like TNF-α and interleukin 1n (45). Second, gingipains can directly degrade components of the intestinal mucus layer and downregulate the expression of tight junction proteins (e.g., ZO-1, TJP-1, OCLN), impairing epithelial barrier function and increasing the risk of a “leaky gut” (40). Impaired intestinal barrier function is a pivotal initiating event that links gut health to oral disease progression. When this barrier becomes permeable, bacterial endotoxins such as LPS enter the systemic circulation. As potent pro-inflammatory molecules, they induce the release of TNF-α and IL-6 and promote reactive oxygen species (ROS) generation, fueling a state of chronic systemic inflammation (**Figure 3**). This persistent, low-grade inflammation “cultivates” a pro-tumorigenic microenvironment. In the context of OSCC, it drives disease progression by causing mucosal cell damage, inducing an immunosuppressive state, and enhancing local angiogenesis—collectively accelerating the malignant transformation of chronically inflamed tissue. Studies have confirmed that gut barrier damage can exacerbate RIOM and increase the risk of tumor recurrence (46). Furthermore, local oral pathogens can amplify this process by modulating immune responses and suppressing anti-tumor immunity, thereby facilitating immune evasion and creating favorable conditions for tumor proliferation and metastasis.

Oropharyngeal candidiasis (OPC), an opportunistic fungal infection caused by *Candida albicans*, is closely associated with local microecological imbalance. Dysbiosis induced by C. albicans has a dual impact: it introduces oral microbial antigens to the gut and promotes the migration of oral-originated Th17 cells to the intestinal tract. These gut-homing Th17 cells can subsequently activate intestinal Th17 cell subsets with oral pathogenic potential, ultimately exacerbating intestinal inflammation. This demonstrates that oral mucosal immune responses are not confined locally but are subject to “compartmentalized” regulation, likely co-modulated by the microbiota of both the oral and gut ecosystems. From the perspective of gut ecology, segmented filamentous bacteria (SFB), key components of the murine gut microbiota, are anaerobic, Gram-positive microbes that primarily colonize the distal ileum (47, 48). They act with remarkable specificity: by directly adhering to the intestinal epithelial surface, SFB potently activate local Th17 cells within the gut. Although SFB colonization is strictly confined to the intestine (49), the Th17 cells they activate can mediate a range of pathophysiological events in extra-intestinal tissues, underscoring the potential for oral-gut microbial crosstalk (50). Animal models further substantiate this gut-oral connection. In a mouse model of periodontitis, the abundance of *Klebsiella* in the gut was significantly increased, and *Klebsiella* from saliva was shown to translocate directly to the intestine. These gut-colonizing bacteria potently induce Th1 cell differentiation and promote the generation of oral pathogen-reactive Th17 cells. These Th17 cells can migrate from oral lymphoid tissues to the gut lamina propria, where they are reactivated by resident *Klebsiella*, thereby intensifying intestinal inflammation—a process that reveals a trans-site inflammatory mechanism: “oral inflammation → microbial translocation → gut immune activation.” Conversely, gut dysbiosis can also predispose to oral diseases. By interfering with the differentiation and function of T cells, macrophages, and dendritic cells, an imbalanced gut microbiota can weaken local immune surveillance in the oral cavity. This is often manifested by a reduction in regulatory T cells (Tregs), which are essential for maintaining immune homeostasis, and an increase in pro-inflammatory Th17 cells. Such immune imbalance exacerbates oral inflammation and can promote the development of OSCC. Th17 cells, a critical subset of T helper cells, primarily exert pro-inflammatory effects by secreting interleukin 17A, interleukin 17F, and the chemokine CCL20. In contrast, Tregs counterbalance this response through a dual mechanism: secreting inhibitory cytokines like IL-10 and TGF-β, and mediating cell-cell suppression via immune checkpoint molecules such as T cell immunoreceptor with Ig and ITIM domains and cytotoxic T lymphocyte-associated antigen 4 (CTLA-4). The functional equilibrium between Th17 cells and Tregs is intimately linked to the chronic inflammatory state during tumorigenesis. However, the precise role of Th17 cells remains contentious: some studies ascribe a tumor-promoting function to them and their associated cytokines, while others suggest they may be pivotal for anti-tumor immunity. In HNSCC, radiotherapy can induce anti-tumor immunity to indirectly kill cancer cells. However, tumors can counteract this by upregulating PD-L1, secreting immunosuppressive factors, and recruiting Tregs and myeloid-derived suppressor cells. Tregs not only inhibit tumor-antigen-specific immune responses but are also recruited to the tumor microenvironment (TME) following radiotherapy, where they attenuate radiation-induced tumor cell death. They mediate immunosuppression through both contact-dependent and independent mechanisms (e.g., sequestering interleukin 2), producing IL-10, TGF-β, and adenosine). Depleting Tregs alone often fails to induce effective anti-tumor immunity due to low infiltration of effector T cells. Radiotherapy can transform poorly immunogenic tumors by exposing neoantigens and promoting the secretion of CXCL9/CXCL10, thereby enhancing immune recruitment. Hypofractionated radiotherapy has been shown to increase the number and function of CD8+ T cells while reducing myeloid-derived suppressor cells accumulation in HNSCC models. A single 10 Gy dose can also significantly boost CD8+ and CD4+ T cell numbers. Critically, combining radiotherapy with Tregs depletion significantly improves tumor response in HNSCC, an effect not achievable by either treatment alone (51). Some studies further associate a high Treg gene signature with reduced overall survival in irradiated patients, although targeting Tregs (e.g., with anti-CD25 antibodies) remains challenging. The mitogen-activated protein kinase (MAPK) signaling pathway is a central regulator of fundamental cellular processes, including proliferation, survival, and apoptosis. Through a cascade of phosphorylation events, it activates downstream targets that govern cell growth, immune responses, inflammation, and stress adaptation (52). The extracellular signal-regulated kinase (ERK) pathway, a core branch of the canonical MAPK cascade, transmits signals from cell surface receptors to the nucleus, where it upregulates the transcription of cyclin D1 to drive cell cycle progression. ERK1 and ERK2 are the primary functional kinases in this pathway, regulating critical events such as cell proliferation, differentiation, migration, invasion, and carcinogenesis by phosphorylating diverse substrates. Aberrant activation of the MAPK pathway is a common feature in cancer, arising from genomic alterations in pathway components or from abnormal receptor tyrosine kinase (RTK) signaling. Both mechanisms lead to sustained MAPK/ERK signaling, creating a permissive environment for tumorigenesis (53). Notably, Pg has been confirmed to promote HNC cell invasion by activating the MAPK/ERK signaling pathway (54).

Simultaneously, various metabolites produced by the gut microbiota, such as tryptophan-derived indole derivatives, serve as crucial signal carriers along the gut-oral axis, modulating host metabolism, immunity, and inflammation. The gut microbiota converts tryptophan into immunoregulatory indole derivatives, which are vital for maintaining immune homeostasis. Gut dysbiosis can disrupt this tryptophan metabolism, leading to abnormal production of these metabolites. This dysregulation can, in turn, exacerbate inflammation and immune dysfunction, ultimately creating favorable conditions for the initiation and progression of OSCC (5). The gut-oral axis plays a significant role in OSCC pathogenesis: gut dysbiosis promotes a systemic pro-inflammatory and pro-carcinogenic state by increasing intestinal permeability, triggering endotoxemia, and sustaining chronic inflammation. Concurrently, oral dysbiosis exacerbates local inflammation, immune suppression, and cellular damage. Together, they act synergistically to drive oncogenesis.

Intervention strategies targeting microbial regulation demonstrate promising clinical potential. Several trials have confirmed that probiotic supplementation can significantly reduce the incidence of severe (Grade 3) OM in patients undergoing radiotherapy (p < 0.05) and help maintain CD4+/CD8+ T-cell ratios (76.59% vs. 52.85%) (55). Supporting this, a study by Jiang et al. involving 99 HNC patients receiving radiotherapy found that a probiotic consortium containing *Bifidobacterium longum*, *Lactobacillus lactis*, and *Enterococcus faecalis* significantly reduced the incidence of Grade 3 OM compared to a placebo. Furthermore, the probiotic treatment helped restore the gut microbiota to a dynamic balance resembling that of healthy controls. Beyond probiotics, antimicrobial peptides (e.g., nisin) offer another targeted approach by selectively inhibiting periodontal pathogens while sparing normal oral cells (6). These collective findings provide a compelling rationale for developing novel comprehensive treatment strategies for HNC centered on the “oral-gut axis.” Future research should, therefore, prioritize elucidating the molecular mechanisms by which this axis coordinately regulates tumorigenesis, with a focus on deciphering complex microbial interactions, host and microbial metabolic reprogramming, and immune microenvironment remodeling. A deeper understanding of this regulatory network will not only furnish a theoretical foundation for new therapeutic modalities but is also expected to enable the bidirectional modulation of oral and gut microbiota through multi-dimensional interventions targeting key network nodes. This refined approach holds the potential to significantly enhance clinical efficacy and improve patient prognosis.

### 4.1 The impact of environmental behavioral factors on the microbiome

Diet and environmental factors have significant regulatory effects on the composition and function of the oral gut microbiota: a high fiber diet is the core of maintaining gut microbiota diversity and health. The complex carbohydrates it contains can become an energy source for specific bacteria through colonic fermentation, and differences in fiber structure can affect the composition and function of the microbiota; A high-fat diet can disrupt the balance of the microbiota, increase endotoxin production, disrupt the intestinal barrier, and induce inflammation and metabolic disorders. Another study shows that using alcoholic mouthwash twice a day can increase the risk of cancer in smokers by more than 9 times, for drinkers by more than 5 times, and for those who never drink, it is almost 5 times. In addition, environmental factors such as antibiotics, smoking, and pollutants can also disrupt the balance of oral gut microbiota, leading to dysbiosis of the microbiota (3).On this basis, interventions such as diet, lifestyle, prebiotics, and prebiotics can optimize the efficacy of immune checkpoint inhibitors (ICI) by regulating the microbiota. Additionally, Mohammad Reza Shafiei confirmed that probiotic therapy can reduce the severity of OM disease. However, while products containing *Bifidobacterium* and *Lactobacillus* can help alleviate the severity of OM, products containing *Streptococcus* are not effective (10).In terms of dietary intervention, strategies such as ketogenic diet and choline supplementation can enhance anti-tumor immune efficacy by leveraging microbial communities and their metabolites; Fructose, high salt, and Mediterranean diet also have immunomodulatory potential, but diet induced changes in microbiota are reversible, and treatment efficacy is influenced by initial microbiota composition and multi system interactions. Therefore, precise treatment plans need to be developed during critical treatment periods; In terms of lifestyle intervention, fasting and exercise can positively regulate the microbiota to enhance the efficacy of ICI; Probiotics (such as inulin and pectin), active ingredients in traditional Chinese medicine, and specific polyphenolic compounds can target and regulate bacterial communities, synergistically enhancing the efficacy of ICI; Postbiotics (such as SCFAs) can directly regulate host metabolism and immunity, and when combined with anti-PD-1 therapy, can enhance anti-tumor effects. However, the effects of SCFAs on different types of ICI may vary, which may limit the activity of anti-CTLA-4 therapy. The specific mechanism still needs to be further studied (56).

The application of antibiotics can also have an impact on the composition of the gut microbiota. Some antibiotics can enhance the therapeutic effect of ICI by targeting and clearing carcinogenic bacterial strains, regulating the structure of gut microbiota. For example, vancomycin can enhance the tumor suppressive effect of CTLA-4 inhibitors by adjusting the proportion of microbiota; Antibiotics targeting harmful microorganisms can also reprogram the immunogenicity of TME. Currently, relevant clinical trials are actively exploring the potential application of combination therapy with antibiotics and alpha PD-1. From the mechanism of action analysis, antibiotic induced dysbiosis can cause excessive proliferation of pathogenic bacteria, reduce the number of beneficial bacteria such as Bifidobacterium and Lactobacillus, and damage the integrity of the intestinal barrier, downregulate the expression level of mucosal surfactant cell adhesion molecule-1 (MAdCAM-1), and promote the migration of T cell subsets with immunosuppressive function to TME; In addition, this dysregulated state can inhibit the signaling pathway of anti-tumor T cells, impair antigen presentation function and effector T cell activity, ultimately weakening the therapeutic effect of ICI. On the contrary, precise use of antibiotics can target the clearance of tumor promoting bacteria such as Fn, promote the release of microbial neoantigens, and activate CD8+T cell-mediated anti-tumor immune responses, while inhibiting immune suppression related signaling pathways. In addition, smoking and alcohol consumption can significantly reduce the relative abundance of Actinobacteria in the gut of patients with OSCC. However, there is a therapeutic paradox in antibiotic induced gut microbiota imbalance, and its impact on ICI efficacy depends on the type and timing of antibiotic administration. For the use of antibiotics in HNC patients, targeted dosing guidelines need to be developed in the future to achieve a balance between infection control and immunotherapy efficacy (56).

### 4.2 The impact of HPV on microbiome, immune microenvironment, and prognosis

Chronic persistent HPV infection is an important cause of HNC, and its infection characteristics and carcinogenic mechanisms have clear specificity. The overall positive rate of HPV DNA in HNC patients is 15.7%, with HPV16 being the main infectious type, accounting for 88.5%; There is a significant difference in HPV infection rates among different subtypes of HNC, with OSCC having the highest infection rate (66.7%), followed by laryngeal cancer (16.7%), nasal cancer (12.5%). No HPV infection was detected in pharyngeal squamous cell carcinoma (57). From the perspective of pathogenic types, HPV positive HNC caused by HPV16/18 accounts for about 85%, while the remaining 15% is caused by HPV33, 35, 52 and other types, HPV6, 11. A total of 9 strains, including 16, cause 90% of HNC cases (58).

The carcinogenic core of HPV lies in the synergistic effect of non-structural proteins E5, E6, and E7: E5 has anti-apoptotic function and can overactivate the epidermal growth factor receptor signaling pathway to stimulate cell proliferation; E6 binds to the tumor suppressor protein TP53 through the ubiquitin protein ligase E6AP/E3A and mediates its proteasomal degradation; E7 can inhibit retinoblastoma proteins and both can bypass cell cycle checkpoints. In addition, E6 can inhibit the interferon transcription factor IFR3, E7 can inhibit Toll-like receptor 9 in immune cells, jointly weakening the body’s immune response, and E6 and E7 can upregulate telomerase TERT expression to achieve cell immortality. Smoking, immune suppression, and other viral infections are key auxiliary factors that promote the progression of HPV positive HNC. Smoking can downregulate the tumor suppressor gene miRNA-133a-3p, upregulate epidermal growth factor receptor and Hu antigen R, increase the probability of oral HPV infection, damage immune mediators, and promote the integration of HPV DNA with host DNA; According to reports, the risk of HPV infection in immunocompromised populations such as HIV/AIDS patients is three times higher than that of the general population, and they are more prone to multiple co infections (59).

High risk HPV infection is a powerful factor in predicting the specific survival rate of non OSCC patients. Studies have shown that HPV positive OSCC patients are more sensitive to chemotherapy and radiotherapy, with overall survival (OS) and disease-free survival (DFS) better than HPV negative patients. This conclusion has also been confirmed by researchers at Vanessa B. WOOKY (60).

HNC patients exhibit significant dysbiosis of the oral microbiota, which is closely associated with HPV infection status, disease progression, and prognosis. Differentially enriched microbiota are associated with HNC subtypes, T staging, survival outcomes, recurrence risk, HPV infection status, and smoking history. Specifically, the enrichment of symbiotic genera including *Corynebacterium* and *Phocaeicola* is negatively correlated with the abundance of pathogenic genera such as *Clostridium*.; High risk HPV infection is associated with the aggregation of Porphyromonas in OSCC and non OSCC; The enrichment of Clostridium in OSCC tissues is significantly correlated with non-smoking, early T and N staging, and better 3-year disease-specific survival in patients (57). In HPV positive tonsil cancer-related specimens, *Pseudomonas* aeruginosa is a Gram-negative aerobic bacterium unique to all cancer-related specimens (metastatic/non metastatic tonsil tissue, metastatic lymph node tissue). Among them, the metastatic group specimens only contain *Shigella*, *Staphylococcus aureus*, and *Neisseria gonorrhoeae*, and the number of Gram-negative bacteria in cancer patient samples shows an increasing trend (61). There is a significant difference in the beta diversity of oral microbiota between HPV positive and negative HNC patients and healthy controls, while there is no difference in alpha diversity. *Streptococcus* is the dominant bacterium in all three groups, and the dominant bacteria in the HNC and HC groups are *Streptococcus mucilaginosus* and *Haemophilus parainfluenzae*, respectively; At the level of phylum, family, and genus, the abundance of phyla such as Zygomycota and Bacteroidetes increased in HPV positive samples, while the abundance of phyla such as Proteobacteria decreased. The abundance of genera such as Firmicutes and Treponema increased, while the abundance of genera such as *Neisseria* and *Lactobacillus* decreased. Additionally, the abundance of the genus *Treponema* and its related species increased significantly in adjacent normal oral tissues of HPV-positive individuals. In addition, the relative abundance of 26 periodontal disease related bacteria in the HPV positive HNC group was higher than that in the HPV negative group. Invasive bacteria such as filamentous bacteria can coexist with HPV in oral lesions, suggesting that the two may have a synergistic carcinogenic effect; *Spiroplasma* contains virulence factors such as chymotrypsin, which may promote epithelial tissue invasion. *Clostridium* can mediate tumor proliferation by activating chemokines, and its enrichment helps improve the prognosis of OSCC patients (58). The tumor microenvironment and clinical characteristics of HPV positive HNC also exhibit uniqueness, and there are significant differences compared to negative HNC. Dysbiosis of oral microbiota is involved in the pathogenesis of OSCC, and its complex interactions with risk factors such as HPV infection and smoking play an important role in the occurrence and development of HNC. Clarifying these associations can provide important basis for the study of HNC pathogenesis, early diagnosis, and targeted treatment plan formulation.

### 4.3 Microbial regulation of HNC immune therapy response

With the development of tumor immunology, ICI have become an important direction for HNC treatment, and the composition of microorganisms is closely related to the efficacy of ICI therapy, *Faecalibacterium*,Specific bacterial populations such as *Bifidobacterium* and *Akkermansia muciniphila* can enhance the efficacy of ICI by inducing T cell differentiation, regulating the cytokine environment, or indirectly shaping tumor immune responses through their metabolites; Antibiotics can disrupt the diversity and structure of the microbiota, causing dysbiosis and ultimately reducing the effectiveness of ICI. Previous studies have confirmed that various microbial communities and their metabolites (such as polysaccharides A, inosine, deamination tyrosine, SCFAs, etc.) can enhance anti-tumor immune responses by activating dendritic cells, NK cells, and T cells, enhancing antigen presentation, and promoting tumor antigen-specific T cell expansion. Some strains’ metabolites can also improve CAR-T cell efficiency (62).

Griffin’s team’s research indicates that the anti-tumor enhancing effect of peptidoglycan hydrolase SAGA secreted by *Enterococcus* is achieved through targeted activation of NOD2 receptors; Probiotics such as *Bifidobacterium* and *Staphylococcus aureus* regulate through multiple pathways, including promoting dendritic cell activation, inducing interleukin 12 secretion, activating the cyclic GMP-AMP synthase/stimulator of interferon genes signaling pathway, and ultimately enhancing CD8 ^+^ T cell function; In addition, gut probiotics can stimulate immunoglobulin A synthesis and secretion at the mucosal immune level, not only enhancing mucosal immune surveillance efficacy, but also accurately regulating the transport process of symbiotic bacteria; *Lactobacillus*, *Bifidobacterium* and other strains can degrade tryptophan into indole derivatives, thereby enhancing the killing effect on tumors and improving the clinical efficacy of ICI; Jia et al. found that the indole-3-propionic acid co-produced by *Lactobacillus Johnson* and *Clostridium* sp*orogenes* can enhance the efficacy of anti-PD-1 by regulating T cell stem like characteristics. Metabolites of gut microbiota are also key mediators in regulating immune therapy sensitivity. SCFAs (acetate, propionate, butyrate) can regulate immune function by binding to G protein-coupled receptor or inhibiting histone deacetylase. In addition, butyrate can upregulate/CD28 expression and enhance the efficacy of alpha PD-1 through the histone deacetylase inhibition pathway; Meanwhile, butyrate can also induce Tbx21 transcriptional inhibition of PD-1 and alleviate CD8 ^+^ tumor-infiltrating lymphocyte depletion (56).

The predictive value of microbiota on PD-1/PD-L1 inhibitor response remains controversial: the largest clinical oriented study, CheckMate141, analyzed saliva samples and did not find a significant association between oral microbiota and PD-L1 inhibitor efficacy, but multiple preclinical studies still reveal the potential regulatory role of microbiota. Hu et al. found that the presence of *Luteibacter*, *Flammeovirgo*, and *Chlamydia trachomatis* is significantly correlated with the total number of T cell receptors, clones, and CD8 ^+^ T cell infiltration in HNC patients. The mechanism may be mediated by upregulating the levels of chemokines in TME, promoting T cell recruitment and infiltration, and mediating the response to ICI (63).

In the research system of the interaction between microbiota and head and neck tumors, the mining and validation of microbial toxicity prediction factors have been one of the core directions of clinical translational research in recent years. The Yuchao Li team collected saliva, subgingival plaque, and tongue dorsal mucosa samples from patients with gingival squamous cell carcinoma (GSCC) through a system for microbiota analysis. The results showed that periodontal pathogens were enriched in GSCC lesions and related areas. Meanwhile, the high expression level of atorvastatin in saliva and abnormal biosynthesis of endotoxins may significantly increase the risk of periodontitis in patients. *In vitro* cell model experiments have confirmed that persistent infection of *Gingival pseudomonas* can effectively promote the proliferation, migration, and invasion ability of human oral epithelial cells, and induce their malignant transformation (64).

Another study found that the saliva microbiome of OSCC exhibits a phenotype with significantly increased alpha diversity and specific beta diversity characteristics. The specimen is enriched in genera such as *Streptococcus*, *Lactobacillus*, *Prevotella*, *Trichomonas* and *Haemophilus*. Among them, *Lactobacillus* is significantly more abundant in the saliva of oral squamous cell carcinoma patients than in the control group, and is more enriched in advanced T and N stage patients. In contrast, the dominant genera in healthy control samples are *Actinobacteria* and *Corynebacterium* , *Prevotella* and fine haired bacteria are often associated with tumor suppression or good prognosis: *Actinobacteria* have low virulence and are core bacterial species in healthy populations; *Corynebacterium*, as a dominant symbiotic bacterium in healthy saliva, is associated with high abundance and low risk of HNC; The abundance of filamentous bacteria decreases with the progression of oral mucositis, and high abundance within the tumor corresponds to higher patient survival rates (65).

The Soyoung Kwak team found a correlation between 13 types of oral bacteria and the risk of HNC through oral microbiota analysis. Among them, four strains of the Proteobacteria phylum (*Acinetobacter baumannii*, *Pseudomonas aeruginosa*, *Klebsiella pneumoniae*, and *Pasteurella multocida*) are associated with a reduced risk of HNC; The red and orange bacterial pathogen complexes in the oral cavity are moderately positively correlated with the risk of HNC. In addition, no correlation was found between the detected fungal species and the risk of HNC (66).

## 5 Conclusions

HNC remains a significant global health burden, with its prevalence increasingly linked to modern lifestyle factors. While traditional risk factors are well-established, the oral and gut microbiota have emerged as pivotal contributors to HNC pathogenesis. The “oral-gut axis” represents a dynamic, bidirectional communication network that fundamentally influences cancer initiation, progression, and therapeutic response. This paradigm provides a novel microecological perspective for re-examining HNC, opening new avenues for prediction, prevention, diagnosis, and treatment.

The interplay between oral and gut microbiota, facilitated by the oral-gut axis, plays a critical role in HNC development. Advances in technologies such as high-throughput sequencing and Mendelian randomization have deepened our understanding of the composition, diversity, and dynamic changes within these microbial communities. The core insight from this field is the departure from the traditional view of “oral diseases as localized entities,” instead revealing the systemic impact of gut microbiota on HNC through “cross-boundary” interactions. This holistic, microecological perspective offers a more coherent explanation for the multifactorial etiology of HNC—such as the elevated incidence of oral squamous cell carcinoma in immunocompromised individuals. Furthermore, it unveils new targets for precision medicine, suggesting that early intervention in processes like tryptophan metabolism or biofilm formation could potentially block carcinogenesis.

Despite promising advances, several challenges persist. First, current research often focuses on single microbial species (e.g., Fn, Pg), lacking a systematic understanding of the comprehensive microbial interaction networks. Second, the clinical translation of findings is hindered by the insufficient validation of microbial biomarkers in large-scale cohorts and the absence of standardized intervention protocols (e.g., probiotic strains, timing) for modulating the oral-gut axis in HNC patients. Third, most mechanistic insights are derived from *in situ* HNC mouse models, which cannot fully recapitulate the complex human context shaped by diet, genetics, and lifestyle.

In summary, the interaction network of oral and gut microbiota has unveiled a new frontier in head and neck cancer research. Although mechanistic and translational challenges remain, the oral-gut axis holds great promise as a breakthrough target for the precise diagnosis and treatment of HNC, offering new hope for improving patient outcomes. Future progress will depend on close multidisciplinary collaboration across oncology, microbiology, immunology, and dental medicine to translate these foundational discoveries into clinical practices that benefit HNC patients.
