# Correction: Cross-disciplinary communication between oral and gut microbiota in head and neck cancer

**DOI:** 10.3389/fonc.2026.1805307

**Published:** 2026-03-04

**Authors:** Xinhua Lin, Hanbin Qin, Zhonglu Liu, Xin Zhao, Xuexia Liu, Hua Zhang

**Affiliations:** 1 School of Clinical Medicine, Shandong Second Medical University, Weifang, China; 2 Department of Otorhinolaryngology, Head and Neck Surgery, Yantai Yuhuangding Hospital, Qingdao University, Yantai, China; 3 Shandong Provincial Clinical Research Center for Otorhinolaryngologic Diseases, Yantai, China; 4 Shandong Provincial Key Laboratory of Neuroimmune Interaction and Regulation, Yantai, China; 5 Shandong Engineering Research Center for Precision Diagnosis and Treatment of Airway Diseases, Yantai, China; 6 Yantai Key Laboratory of Otorhinolaryngologic Diseases, Yantai, China; 7 Yantai Clinical Research Center for Otorhinolaryngologic Diseases, Yantai, China; 8 Shandong Stem Cell Engineering Technology Research Center, Central Laboratory, Affiliated Yantai Yuhuangding Hospital of Qingdao University, Yantai, China

**Keywords:** gut microbiota, HNC, oral microbiota, oral-gut axis, tumor

There was a mistake in **Figure 1** as published. **Figure 1** has not been updated to the final version. The corrected **Figure 1** appears below.

**Figure 1:**
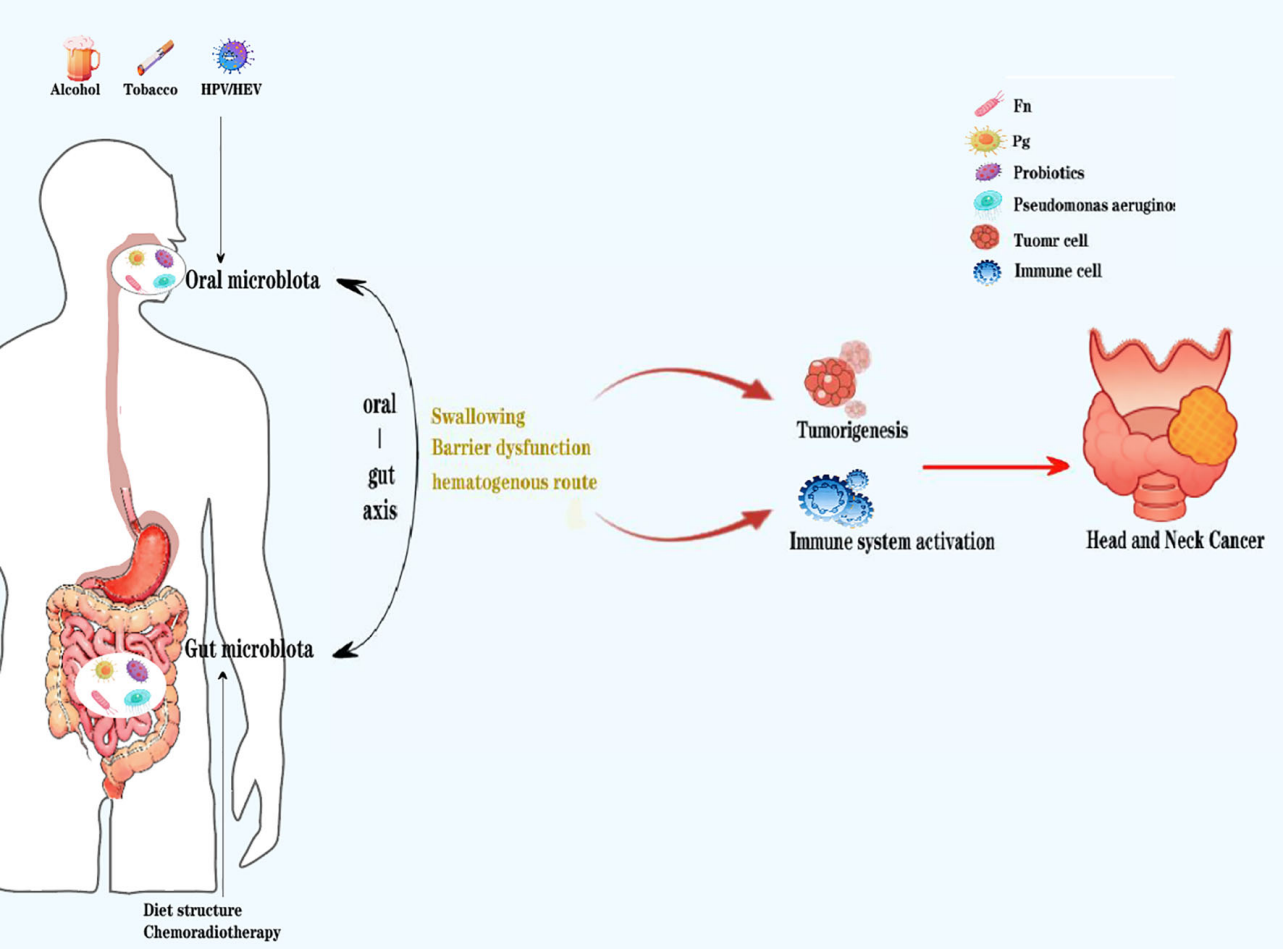
When the oral and gut microenvironment changes due to external factors (such as smoking, alcohol consumption, HPV/HIV virus, dietary structure,chemotherapy, etc.), the microbial community structure is prone to imbalance, mainly manifested as an increase in pathogenic bacteria such as Pg and Fn, and a decrease in beneficial bacteria such as probiotics. Due to damage to the oral and intestinal mucosal barrier, the microbiota undergoes significant enhancement of local oral and gut microbiota migration through swallowing and blood exchange, thereby disrupting the homeostasis of the systemic immune microenvironment, promoting tumor cell growth, and ultimately creating conditions for the occurrence and development of head and neck tumors.

There was a mistake in the captions of **Figure 2** and **Figure 3** as published. The term “oral cavict” is a typographical error. The corrected captions of **Figure 2** and **Figure 3** appear below.

**Figure 2:**
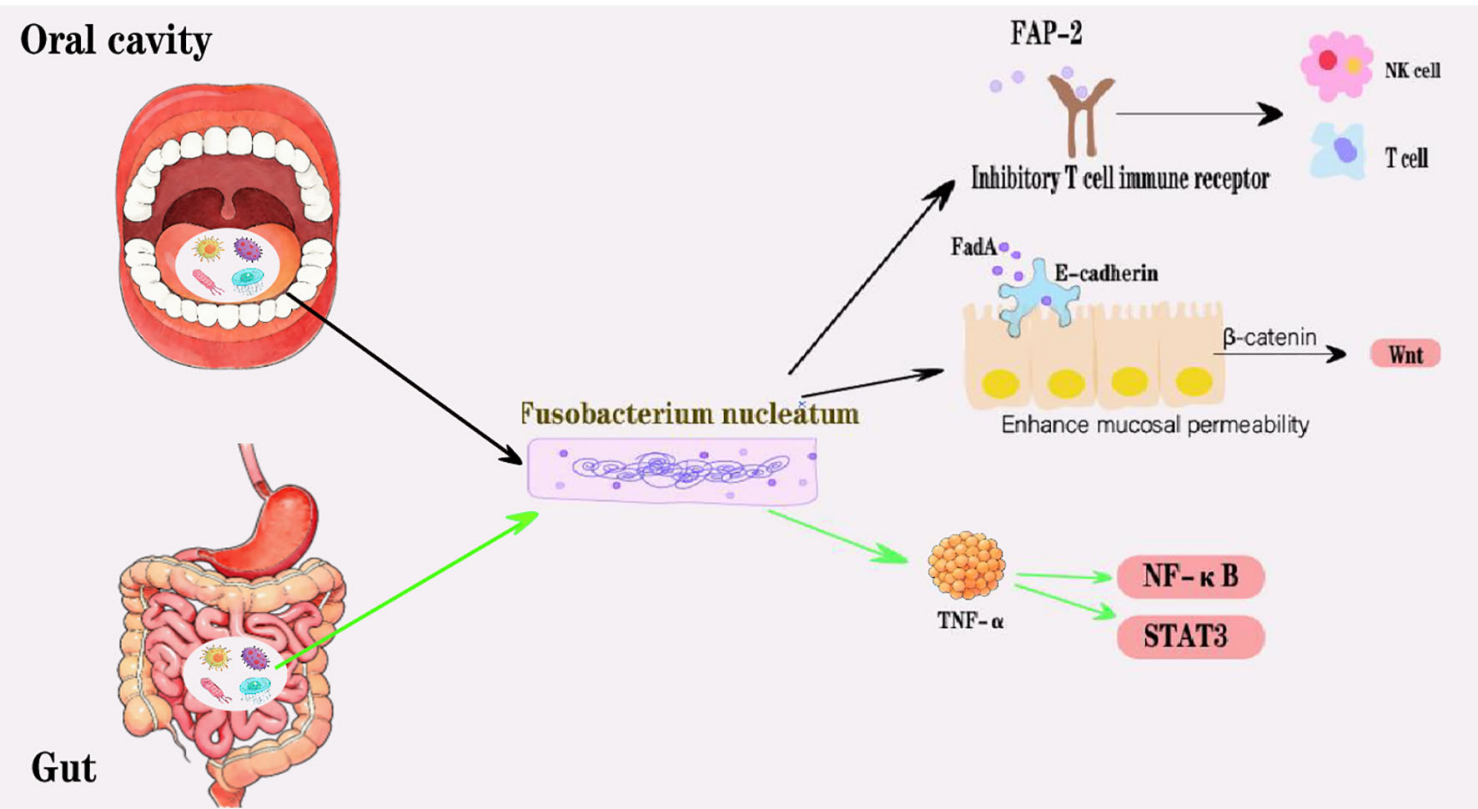
In the oral cavity, Fn’s adhesion protein FadA can bind to E-cadherin on the surface of epithelial cells and inactivate it, thereby enhancing mucosal permeability. Simultaneously, the intracellular free breeacell will also increase, activating the Wnt signaling pathway. Furthermore, Fn proteins (such as FAP-2) can block and reduce the activity of NK cells and T cells by binding to inhibitory T cell immune receptors. In intestinal tissues, Fn can promote the production of TNF-uc activate the NF-iv signaling and STAT3 signaling pathways. Fn may achieve the invasion and metastasis of HNC through the above mechanism.

**Figure 3:**
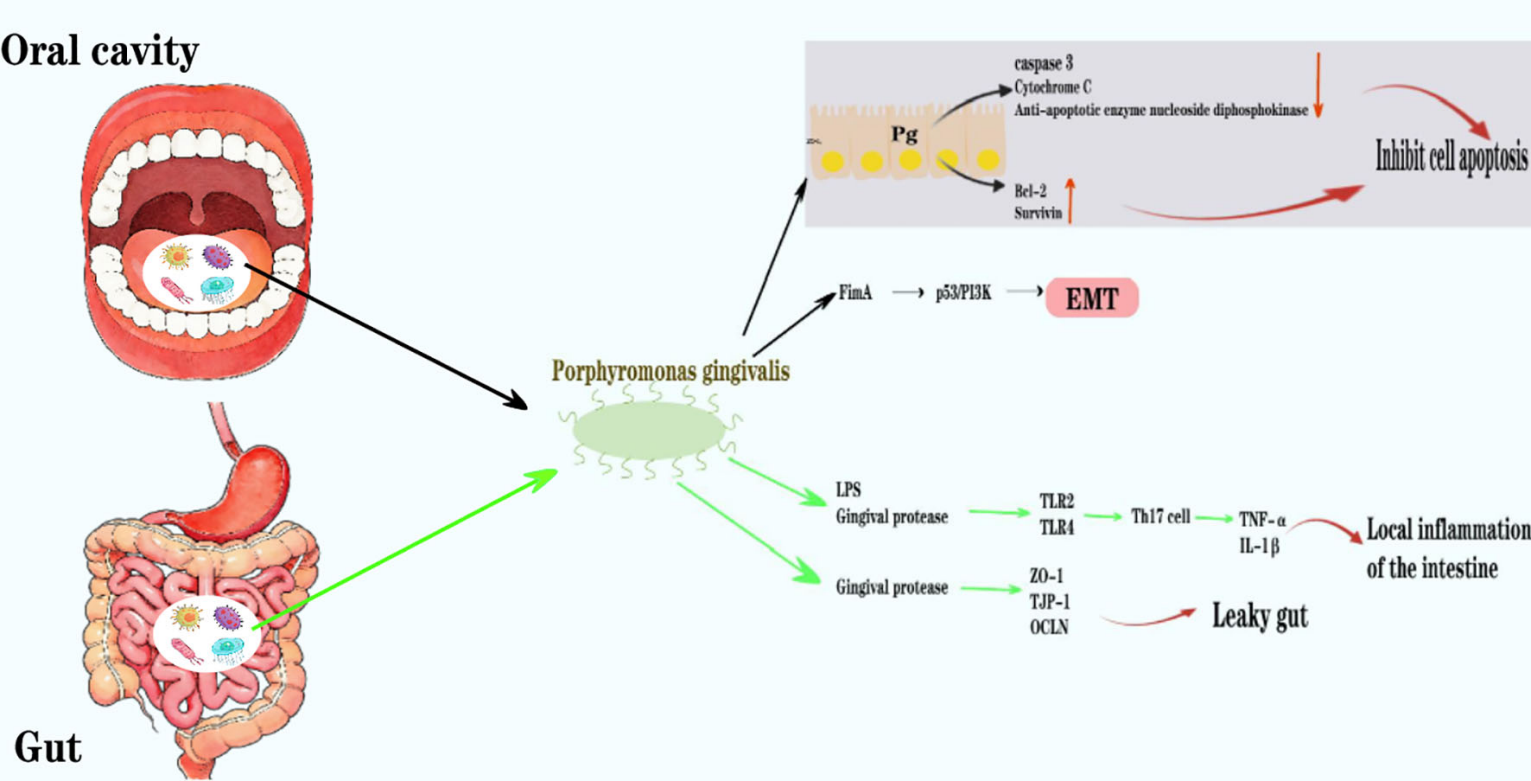
In the oral cavity, Pg inhibits apoptosis through multiple mechanisms, including inhibiting P cell pigment C, downregulating caspase 3 activity, secreting anti-apoptotic enzyme nucleoside diphosphokinase, and upregulating microRNA; Pg can induce epithelial-mesenchymal transition and primarily affects p53, PI3K, and cell cycle protein pathways through its FimA adhesion molecule, thereby accelerating the cell cycle progression. In the intestine, components of Pg such as LPS, gingipain, and extracellular vesicles induce the differentiation of helper T cell 17 (Th17 cells) by stimulating TLR2 and TLR4, thereby promoting the expression of pro-inflammatory factors such as tumor necrosis factor (TNF) and interleukin 1n. This leads to local inflammation in the intestine; the gingipain it produces can directly degrade components of the intestinal mucus layer, disrupting the integrity of the mucus barrier, and simultaneously downregulating the gene expression of tight junction-related proteins (such as occludin 1, tight junction protein 1/TJP-1, and occludin/OCLN) through cleavage, resulting in impaired intestinal epithelial barrier function and increased risk of “intestinal leakage”.

The original version of this article has been updated.

